# Tackling challenges of global health electives: Resident experiences of a structured and supervised medicine elective within an existing global health partnership

**Published:** 2017-04-20

**Authors:** Michelle Tubman, James Maskalyk, David Mackinnon, Raghu Venugopal, Elayna Fremes, Lisa M Puchalski Ritchie, Aklilu Azazh, Megan Landes

**Affiliations:** 1 University of Toronto, Ontario, Canada; 2 St. Michaels Hospital, Toronto, Ontario, Canada; 3 Division of Emergency Medicine, Department of Medicine, University of Toronto, Ontario, Canada; 4 Division of Emergency Medicine, Department of Family and Community Medicine, University of Toronto, Ontario, Canada; 5 University Health Network, Toronto, Ontario, Canada; 6 Toronto Addis Ababa Academic Collaboration in Emergency Medicine, University of Toronto, Ontario, Canada; 7 College of Health Science, Addis Ababa University, Addis Ababa, Ethiopia

## Abstract

**Background:**

The Toronto-Addis Ababa Academic Collaboration in Emergency Medicine (TAAAC-EM) deploys teaching teams of Canadian EM faculty to Addis Ababa to deliver a longitudinal residency curriculum. Canadian trainees participate in these teams as a formally structured and supervised elective in global health (GH) and EM, which has been designed to enhance the strength of GH electives and address key challenges highlighted in the literature.

**Methods:**

The purpose of this qualitative study was to identify, describe, and evaluate strengths and weaknesses of this elective in relation to its purposeful structure. Residents who completed the elective were invited to participate in face-to-face interviews to discuss their experiences.

**Results:**

The findings show that the residents both chose this elective because of its purposefully designed features, and that these same features increased their enjoyment and the educational benefit of the elective. Supervised bedside teaching, relationships shared with Ethiopian residents, and the positive impact the experience had on their clinical practice in Canada were identified as the primary strengths

**Conclusion:**

Purposeful and thoughtful design of global health electives can enhance the resident learning experience and mitigate challenges for trainees seeking global health training opportunities.

## Introduction

Global health electives are increasingly acknowledged as a valuable educational experience for medical trainees in North America.^1^ While global health can have multiple meanings, we accept the definition of the Office of Postgraduate Medical Education at the University of Toronto that global health is a conceptual framework to understand how expressions of health and illness are impacted by underlying determinants to affect world populations and agree that these electives occur in three main settings: in another country (predominantly low or middle income), within an aboriginal setting in Canada, or working with another local vulnerable population within Canada (such as refugee populations).^2^,^3^

Previous studies have shown global health electives to have several benefits for trainees including enhancing knowledge (e.g., cross-cultural issues, tropical medicine, and public health) and skills (e.g., problem solving, clinical examination), increasing their understanding of health systems and collaborative partnerships, and fostering of attitudes and values embedded in health equity (e.g., interest in serving underserved populations).^4^,^5^,^6^

However, studies also highlight key challenges in the way these electives are conducted. Many global health electives are informally arranged by medical trainees themselves and as such residents often do not receive pre-departure training, preparation for practice in these settings, nor formal debriefing on return.^3^,^6^ Furthermore, a lack of supervision in the host country, undefined roles and responsibilities for the trainee, and unclear educational objectives jeopardize the educational value of such experiences.^3^ Finally, through these informal training structures we risk placing the burden of educating our Canadian trainees on already strained health systems and educational institutions in low-resource settings.^7^ These challenges highlight issues of equity in global health education.

### Description of TAAAC-EM global health elective

The TAAAC-EM global health elective for Canadian residents was purposefully designed to mitigate these key issues often found in global health electives for post-graduate trainees, with a focus on providing adequate preparation, an educationally structured and supervised elective experience, and appropriate mechanisms for debriefing.

The TAAAC-EM global health elective operates within the partnership established by the University of Toronto (UofT) and Addis Ababa University (AAU) in 2010, to support Ethiopia’s first emergency medicine residency training program. The aim of TAAAC-EM is to design and implement an emergency medicine residency training program in Ethiopia, which will address key issues in health human resources shortages and support the development of vital emergency medicine systems. To assist AAU in this goal, teaching teams from U of T, including two faculty from the Divisions of Emergency Medicine and one senior resident, travel to Addis Ababa three times a year for one month. TAAAC-EM accepts only senior level trainees (i.e. FRCP Year 3–5 or CCFP-EM residents) to ensure the resident has adequate skills to participate in a teaching capacity and to practice in a high acuity medical environment. These teams undergo a rigorous selection process (including written application, letters of reference, and interview), and then participate in three pre-departure sessions (one on the partnership, Ethiopian culture, environment and health care systems, one on trip logistics including health and safety issues, and one on educational program implementation and curriculum planning). Each team is organized to provide a section of a locally relevant three-year curriculum via large group and bedside teaching throughout the month and are also prepared to participate in other facets of the program, such as research or ultrasound training. Upon return, the team undergoes one collective and one individual (on a needs basis) debriefing session with the TAAAC-EM leadership.

In addition to addressing the issue of adequate preparation for participating in a global health elective as above, the TAAAC-EM global health elective for Canadian residents was purposefully designed to address key issues around educational structure and supervision. First, trainees meet separately with our TAAAC-EM leadership team to define specifically their educational objectives for the month. These objectives are tailored to trainee interests in aspects of global health practice and have included administration, research or enhanced clinical skills such as bedside ultrasound in low-resource settings. One U of T teaching faculty on each team is then appointed as the specific supervisor of this resident. The majority of U of T teaching faculty have experience practicing in this or similar settings. The faculty are present every day in the ED in Addis with the primary goal of teaching Ethiopian residents, but also of directly supervising the Canadian trainee. This removes the burden of educating our trainees from our host institution and aims to increase the educational value of the elective. U of T faculty provide a mid-term and a final evaluation of the resident using standardized clinical elective evaluation forms from U of T. The faculty provide role-modeling of EM practice in this setting, support for the resident with debriefing the experience (including difficulties with clinical cases or emotional reactions), and any additional mentorship that is specifically related to the trainee’s educational objectives such as exploring global health careers, leadership, simulation or ultrasound.

The primary goal of this study was to describe and evaluate the experience of Canadian residents during the Toronto-Addis Ababa Academic Collaboration in Emergency Medicine (TAAAC-EM) elective in global health focusing on three key challenges identified in the literature: lack of preparation and debriefing, educational structure, and supervision. Secondary goals included describing ways in which the TAAAC-EM resident elective influenced clinical practice of trainees on their return home and their future desire to engage in global health.

## Methods

Senior emergency medicine residents at the U of T who participated in the TAAAC-EM elective between 2011 and 2015 were invited to participate in the study via email. Written consent was obtained from each participant. The study team consisted of one resident who had experience with global health and qualitative research, but had not participated in in this specific elective, and several EM faculty members who are affiliated with the TAAAC-EM program. Ethical approval for this study was granted by the Research Ethics Board at the University Health Network in Toronto.

Data were collected by a single team member via face-to-face interviews. All interviews followed a semi-structured interview guide developed by the study team. The interviews were digitally recorded and transcribed verbatim by an independent party. Each interview took 20 to 30 minutes to complete.

A single researcher coded the transcripts. Data were analyzed using an inductive thematic analysis, where data were coded first into sub-themes and then further divided into categories to identify the salient features of the experience of participants in the elective. The study team composition ensured both inside and outside perspectives informed this analysis.

## Results

All nine residents who completed this elective at the time of study were invited to participate; six participants were included. Five participants were in their third postgraduate year at the time of the elective, and one participant was in their fifth postgraduate year. Participants were interviewed approximately one year after they participated in the elective.

Five of the six participants had previous experience in global health, and all wanted to use this elective as an opportunity to explore the integration of emergency medicine and global health. Several residents described suboptimal experiences in their prior global health electives related to misunderstanding the goals of the elective, lack of supervision during the elective, and feeling unable to contribute at their level of training. Given these prior experiences, residents were motivated to participate in the TAAAC-EM elective as they believed its structured (including preparation, logistical support and debriefing) and supervised approach would provide a more appealing professional and personal opportunity.

In particular, the supervised nature of this elective was repeatedly mentioned as increasing their comfort level in practicing safely and teaching in this environment as there was a direct mechanism for feedback:

Having assigned tasks, and also [to] have supervision from our faculty on site, I think the biggest benefit was because there was direct feedback…the fact that I got feedback from people who already had experience in that setting was important to me. (P06)

While the experience of daily supervision was clearly a benefit cited by all residents, and most felt treated as equal collaborators on the U of T teaching team, some reported experiencing disparities in levels of autonomy permitted by different U of T faculty. Residents felt their structured role in delivering the large group sessions was clear, however these discrepancies in autonomy arose primarily in clinical bedside teaching and practice.

Several of the participants were in their third year of postgraduate training, which marks their first year as a senior resident in Canadian emergency medicine training programs. These residents described feeling overwhelmed by their new role as a senior resident in Canada, and that this feeling was amplified in Ethiopia. Despite these uncertainties, all residents felt that the expectations of both the supervising Canadian faculty and their Ethiopian counterparts were completely appropriate to their level of training, and no resident felt pressure to perform beyond their capabilities.

Two further unique strengths of this elective emerged from our analysis: the value of participating in bedside teaching for the trainees and the collaboration between Ethiopian and Canadian trainees. First, the participants universally identified the clinical teaching they gave at the bedside as the most valuable aspect of the elective, for both their own education, and their personal enjoyment of day-to-day duties. All residents reported a desire to take on more of a teaching role at the bedside, rather than simply observing, for two main reasons: the keen interest of the Ethiopian residents to learn increased their satisfaction of teaching and the bedside teaching around clinical cases increased confidence in clinical practice. Trainees noted the value in the diversity, volume and severity of clinical cases during bedside teaching as challenging their problem solving and critical thinking skills.

The participants also unanimously identified camaraderie with the Ethiopian residents as a unique highlight of the elective. They reported multiple times that the Ethiopian residents were role models for patient advocacy. The enthusiasm and strong motivation of the Ethiopian residents to learn also renewed motivation in the Canadian residents, and made their teaching role increasingly more appealing. The Canadian residents all mentioned the resourcefulness of the Ethiopian residents and how much they learned from them.

We aren’t the Canadian doctors who will teach you everything…we are a part of you, we’re just like you, and we’re learning from you. (P01)

All of the participants made note of impromptu teaching sessions. They identified this as an extremely valuable part of the experience. Socializing outside of the hospital added to their relationship with the Ethiopian residents.

I felt like we formed a strong bond and we’re still in touch, having since then participated in various video teleconferencing sessions. (P06)

Finally, trainees were asked to reflect on how this experience had or would affect their future practice in Canada including their career decisions. Three themes emerged. First, they identified an increased awareness of a scarcity of resources as all participants pointed out the challenges of practicing medicine in a resource-poor setting, and the creativity they witnessed from Ethiopian residents in overcoming the scarcity. Many also commented on how the experience of being in this setting affected their resource allocation and medical decision-making in Canada.

Watching how much you can do with how little….there are certain things you know you have to intervene on, and certain things you don’t. And I think here when we have a lot of resources sometimes those priorities get kind of boggled. (P03)

The second theme that emerged was increased confidence in the management of critical patients. All participants mentioned seeing more high acuity cases in the Ethiopian ED than what they were accustomed to in Canada. These experiences improved their clinical management skills and enhanced their patient-centred approach to critical care and end of life care patients even back in Canada.

I think it’s helped me be calm…in the face of stress with unwell people….I don’t know if I would have had those opportunities otherwise. (P05)

Third, participants were asked to describe the effect the elective had on their future career planning. All participants felt that their elective in Ethiopia solidified an interest in global health. They also felt that prior to Ethiopia their interest was fueled by a desire for adventure or international experience, but now and in the future if they were to engage they would seek work that was mutually beneficial for themselves and the host country. Some stated that it will likely be difficult to incorporate field work into future work, due to both family and professional commitments. Of those who do hope to continue working in global health, all stated a preference for projects that they believed would be responsible and sustainable.

## Discussion

In an era of growing interest in global health education in academic medical institutions and an increasing number of medical trainees participating in global health electives, providing effective and ethical opportunities for medical trainees is paramount**.**^10^,^11^ The TAAAC-EM global health elective in emergency medicine in Ethiopia for Canadian EM residents has been purposefully designed to provide a structured and supervised educational experience that attempts to mitigate common weaknesses traditionally reported around global health electives. The findings in this study provides some evidence that the residents both chose the elective because of these purposefully designed features and that these features increased their enjoyment of the elective and their overall learning.

Two key features of the design of this elective were explored with the residents: a structured program and a supervised experience. The structure of TAAAC-EM includes multiple focused pre-departure briefings along with dedicated time and mentorship in delineated educational objectives and educational expectations. A recent survey of EM programs in the USA showed that the majority of residents participating in global health electives received no pre-departure training or post-elective debriefing or feedback.^6^ We found that residents felt the structured framework of the elective encouraged their participation, a finding consistent with literature that shows increasing emphasis on the importance of adequate preparation of trainees to participate in global health electives.^2^,^12^,^13^ Furthermore, there is increasing discussion in the literature around global health competency based education and the education principles which should guide trainees in these electives.^14^,^15^ This TAAAC-EM elective is an initial attempt to bring educational objectives and standards in evaluation to EM trainees in our institution.

Additionally, this study identifies that residents felt being supervised by a Canadian emergency physician in Ethiopia encouraged them to feel safe in their practice and to learn in a manner appropriate to their level of training. The intentional design of supervision by Canadian faculty while in Ethiopia was meant to mitigate challenging experiences documented in the literature including trainees being asked to act outside their level of competence and unclear expectations around roles and responsibilities.^16^,^17^ This elective appears to address this issue adequately. One related limitation of the study is that we were not able to assess host institution perceptions that the local physicians did not have to leave their other duties to help train our Canadian residents, which again is another key challenge cited frequently in the literature .^8^,^19^,^20^,^21^,^22^

Ideally, international elective programs should seek long-term sustainable relationships with institutions in low-income countries to ensure a mutually beneficial experience for trainees and host countries.^10^ This approach has been successfully demonstrated through the collaboration between Moi University in Kenya with four western medical schools as well as through a partnership between Australia and host communities in India.^21^,^23^ The long-term relationship that has developed between the University of Toronto and Addis Ababa University, and the collaborative approach to providing emergency medicine training ensures a more sustainable effort and the opportunity for longer-term engagement. This study showed that the relationships that residents formed with Ethiopian residents greatly increased their satisfaction with the elective.

To this end, the Working Group on Ethics Guidelines for Global Health Training (WEIGHT) have developed a set of good practice guidelines for educational institutions that send trainees on global health initiatives.^24^ These guidelines stress the need for structured programs between partners with goal of mutual and reciprocal benefit for all institutions involved thus mitigating the adverse consequences of short-term medical trips. This study highlights that the TAAAC-EM elective, through purposeful design of a structured and supervised elective embedded into a long-term academic institutional collaboration, is addressing these objectives and that this purposeful design led to positive experiences among participants.

### Limitations

The study design does not account for other methods of measuring success of the elective design beyond resident experiences and perceptions. This includes a lack of other kinds of data on outcomes or perspectives from our host institution, including potential harms to the host institution.

Methodologically, only six of nine possible residents consented to participate in the study, limiting both the quality and the quantity of data. It’s possible that given the relationship of these residents to the researchers, there was bias towards commenting only on the positive aspects of the elective. In addition, a single coder analyzed the data, again introducing possible bias in interpretation.

### Conclusion

The TAAAC-EM emergency medicine global health elective was designed to directly address key issues in medical electives abroad identified in the literature. Participating EM trainees confirmed that the design of the elective increased both their desire to participate and enhanced their educational experience in both expected and newly identified ways in the literature. Purposeful and thoughtful design of global health electives can mitigate challenges for trainees in seeking these training opportunities. All academic institutions allowing trainees to participate in medical electives abroad should deeply consider their responsibility in advancing the standards of global health education and commit to thoughtful and supportive methods for their trainees to gain experience in global health. Future evaluations of similar innovative designs for global health electives across different cadres of trainees, types of institutions and partnerships could add valuable lessons to the literature for those looking to develop similar programs. These should include a broader range of perspectives including those from host institutions and from patient populations encountered by global health trainees.
